# Adolescent Endorsement of the “Weak-Not-Sick” Stereotype for Generalised Anxiety Disorder: Associations with Prejudice, Discrimination, and Help-Giving Intentions toward Peers

**DOI:** 10.3390/ijerph17155415

**Published:** 2020-07-28

**Authors:** Holly R. Hanlon, Lorraine Swords

**Affiliations:** School of Psychology, Trinity Research in Childhood Centre, Trinity College, Dublin, Ireland; swordsl@tcd.ie

**Keywords:** stigma, mental illness, clinical anxiety disorders, generalised anxiety disorder, help-giving, adolescence

## Abstract

Stigma, comprising negative stereotypes, prejudice (negative affective reactions) and discrimination towards a member of a particular group, is of increasing interest in the context of mental illness. However, studies examining clinical anxiety stigma are lacking, particularly with regard to generalised anxiety disorder (GAD). There is also a lack of research into adolescent anxiety stigma, despite adolescence being a key period for early intervention for anxiety disorders, and research showing that stigma has been implicated in low rates of help-seeking and problematic peer relationships among adolescents with mental illness. Stigma has also been negatively associated with help-giving responses toward those with mental illness. Initial studies suggest that the ‘weak-not-sick’ (WNS) stereotype may be central to anxiety stigma. The present study aims to examine the endorsement of the WNS stereotype in the context of GAD, and its relationship to prejudice, discrimination, and help-giving responses among adolescents. A vignette-based survey measure was completed by 242 adolescents (74 male, 165 female, and three participants who recorded their gender as “other”) in Ireland aged between 15 and 19 years. The results of the study found that endorsement of the WNS stereotype was significantly associated with higher prejudice and discrimination, as well as lower levels of help-giving intentions. A multiple mediator model is presented showing both a direct relationship between endorsement of WNS and help-giving, and an indirect relationship between WNS and help-giving mediated by the prejudicial components of anger, fear and pity, and discrimination as assessed by desired social distance. This study adds to the limited knowledge base on stigma towards GAD in adolescents and provides a model for how anxiety stigma may relate to help-giving. This has implications for interventions to reduce stigmatising and increase help-giving responses.

## 1. Introduction

### 1.1. Background

Clinical anxiety disorders are among the most prevalent mental disorders, affecting over 200 million people worldwide [1,2]. Those affected experience significant and wide-ranging functional impairment, as well as reduced overall quality of life [3,4,5]. As such, these disorders deliver a major addition to the global disease burden [2,4]. Despite this, anxiety disorders are consistently under-recognised and underdiagnosed, with a correspondingly low treatment rate across conditions [6,7]. Overall, studies suggest that less than a third of those with anxiety disorders reported seeking treatment [8,9], with a long duration, often years, from first onset of symptoms to initial help-seeking being common [7,10]. This is especially true of generalised anxiety disorder (GAD) [11].

GAD is one of the most common anxiety disorders, with a lifetime prevalence of around 5% [12]. Marked by excessive and difficult to control worry and anxiety across multiple life domains, as well as symptoms such as muscle tension, trouble concentrating and fatigue, GAD is particularly likely to take a chronic course, with pervasive negative impacts on both the individual and society [1,13]. For these reasons, along with the long delay between symptom onset and help-seeking, it is of particular interest for early intervention.

Many psychological disorders first emerge during adolescence, with the increasing risk in this age group attributed to many factors, from neurobiological and hormonal changes to the unique psychosocial stressors experienced at this transitional stage of life; including a desire for increased autonomy, peer pressure, and an increase in risk-taking behaviour [14,15]. Adolescence has been found to be a key period for the emergence of anxiety disorders, including GAD [16]. Additionally, the presence of clinical and sub-clinical anxiety symptoms in adolescence is predictive of the presence of those same disorders in adulthood, as well as a number of negative outcomes, from poor overall adjustment, educational underachievement, higher levels of chronic stress and increased rates of substance abuse [17,18,19,20]. However, international research has shown that less than one in five adolescents with an anxiety disorder seek professional help [21]. Adolescence, therefore, represents an important opportunity for early intervention in clinical anxiety disorders.

### 1.2. Weak-Not-Sick: Mental Illness Stigma and Anxiety Disorders

Previous research has found that one major factor implicated in negative outcomes for those with mental illnesses is stigma. In their tripartite model of stigma, Corrigan and Watson [22] define stigma as being composed of stereotypes, prejudice (negative affective reactions) and discrimination towards a member of a particular group. These components are related: people who endorse negative stereotypes toward a particular person or group then experience a negative emotional reaction towards them, such as fear, which, in turn, leads to behavioural discrimination [22]. The tripartite model of stigma is regularly utilised in the context of adolescent mental health research and has been validated in adolescent populations [23].

Mental illness stigma has been reported specifically as being a key barrier to seeking help for mental illness in general, and anxiety specifically, in young people [24,25,26] and may also influence the help-giving responses of young people towards peers who require support with a mental health issue [27], suggesting that stigma not only affects whether a person seeks help, but also impacts the response they receive when they do so. Indeed, previous studies have shown that children and adolescents with mental illnesses experience problems in peer relationships and higher levels of victimisation by peers than other conditions [28].

Mental illness stigma then, is particularly concerning in adolescence both because of the key role that peer acceptance plays in the wellbeing of adolescents in general, and more specifically because peers are often the first port of call for young people experiencing mental health problems, with research consistently showing that adolescents prefer to seek informal help from friends [29,30,31,32]. Comprehensively understanding the nature, expression and maintenance of adolescent stigmatising responses is vital in order to inform the development of effective stigma intervention strategies with a view to improving both treatment-seeking for those with mental illnesses and help-giving responses towards them.

To date, generalised anxiety disorder, and anxiety disorders more broadly, have been relatively neglected in the stigma literature. Findings from studies into stigma towards schizophrenia and depression have emphasised the prevalence of violent, unpredictable and dangerous stereotypes, negative emotional reactions such as fear, and a desire for social distance from people with these conditions [33,34,35]. However, it appears that the violent or unpredictable stereotypes may be less applicable to anxiety disorders [36,37]. Instead, the handful of studies which examine anxiety disorder stigma have found that the “weak-not-sick” stereotype (WNS)—that is, a belief that the person’s symptoms do not reflect a real medical condition and instead reflect personal weakness—may be particularly salient for anxiety disorders, as outlined in a recent review [38]. These findings have also been supported by limited studies in adolescent samples [39,40]. This is important, as the WNS stereotype and perceptions of blame in particular have been associated with increased discrimination, negative attitudes, and a decreased likelihood of seeking appropriate help for mental illness [41,42,43,44].

The majority of the few studies exploring anxiety stigma have either focused on social anxiety disorder or post-traumatic stress disorder (PTSD), or did not provide a breakdown of results by disorder [38,45,46], meaning there is a significant gap in the literature examining generalised anxiety disorder specifically. The extremely limited literature on GAD stigma suggests that stigmatising attitudes toward GAD are common among adolescents [47] but detailed research into the content of that stigma is scarce. Studies examining the delay in treatment-seeking for GAD have found that a major reason given is that people with the disorder tend to dismiss symptoms, or normalise them as everyday stress [11]. If people experiencing symptoms of GAD are minimising or failing to recognise the severity of their own condition, this suggests that the WNS stereotype may also be relevant to GAD. To date, no studies have examined the content of GAD stigma in detail in adolescents, particularly with regard to the role of the WNS stereotype. There is also a distinct lack of research into the relationship between the WNS stereotype and help-giving responses toward peers with anxiety disorders, which is concerning, given the key role that peer support plays for young people experiencing mental health difficulties [29,30,31,32].

### 1.3. The Present Study

The present study aims to address these gaps in the literature, by examining stigma toward GAD in a sample of adolescents, as well as the relationship between GAD stigma and adolescents’ help-giving intentions. It is hoped that this paper will provide increased understanding of the associations between the various dimensions of stigma and young people’s help-giving intentions, which has implications for stigma reduction efforts and future programmes aimed at improving mental health first aid responses. Additionally, it will add to the extremely narrow knowledge base on how adolescents perceive peers with anxiety disorders, GAD in particular. Finally, as previous research has shown that the WNS stereotype may be central to anxiety stigma, the paper will examine this stereotype in particular in the context of GAD, a perspective which is lacking in the literature thus far. Stigmatising responses in general have been found to be consistently higher in males than females with regard to GAD as well as other mental illnesses [48,49] and so the present study will also examine the endorsement of the WNS stereotype across the genders.

As such, the following research questions are proposed: 

Do adolescents endorse the WNS stereotype for hypothetical peers presented with symptoms of GAD? Does endorsement of the WNS stereotype differ according to gender?

Does greater endorsement of the WNS stereotype for adolescent peers with GAD relate to greater prejudice and discrimination and less help-giving intentions?

It is expected based on limited prior research in primarily adult samples [38,39] that a proportion of adolescents will endorse the WNS stereotype toward a hypothetical peer with GAD. It is also expected that the WNS stereotype will be endorsed at higher rates by male participants, in line with previous research which has found males to show higher rates of stigmatising responses than females overall [48,49].

Previous findings, which have shown that negative stereotypes in general are associated with increased prejudice and discrimination [22,23] as well as findings showing that perceptions of personal weakness and blame are associated with increased discrimination [41,43], suggest that endorsement of the WNS stereotype will be associated with greater prejudice and discrimination toward peers with GAD. The question of the relationship between endorsement of the WNS stereotype and help-giving responses is largely an exploratory one, due to the lack of existing research in this area. However, research has shown that higher levels mental illness stigma generally may be negatively associated with help-giving responses [27], tentatively suggesting that if the WNS is endorsed, and is associated with increased prejudice and discrimination, then this may be negatively associated with help-giving responses.

## 2. Methods

### 2.1. Participants

Researchers recruited participants from secondary schools and youth groups across the province of Leinster, Ireland, by contacting the organisations via phone and email, resulting in a sample of 242 adolescents recruited from five secondary schools and one youth group across the province of Leinster, Ireland. Participants ranged in age from 15 to 19 years (mean = 16.5 years, SD = 0.8); 74 participants (30.6%) recorded their gender as male, 165 (68.2%) as female, and three participants recorded their gender as “other”. Participants received no compensation for their participation.

### 2.2. Materials

This study was part of a larger study investigating mental health literacy, stigma and help-giving responses toward a variety of clinical anxiety disorders. For the purposes of this paper, analysis was limited to variables examining stigmatising responses and likelihood to help for generalised anxiety disorder (GAD).

The survey measure consisted of brief demographic questionnaire, followed by a brief vignette depicting a young person showing symptoms of GAD. The vignette was developed in accordance with the Diagnostic and Statistical Manual of Mental Disorders (DSM-5) criteria for GAD [1], validated by six trainee clinical psychologists. Participants were randomly assigned either a male (Sean) or female (Katie) vignette character, in order to control for potential effects of vignette gender on responses. The vignette reads as follows:

*“Katie is in 5th year. She loves reading, and often swaps books with her best friends from school. However, over the course of the last year, Katie has found it difficult to relax, and feels like she cannot sit still. She can’t stop thinking about the future and whether she will do well in her exams and get into her first-choice course in college, even though her exams are over a year away. When she sits down to study in the evenings she finds it difficult to concentrate on the work, and her teachers have noticed that she often seems* *distracted during class. Her friends and family have started to notice how tense she is, often about little things. When her mother is late home from work one day, Katie finds herself imagining the worst, that her mother has been in a car accident. She knows that the traffic is heavy and tries to relax, but she can’t stop worrying until her mother gets home safely. Her parents have also noticed that she has been very short-tempered lately, getting angry and slamming doors around the house. She doesn’t even enjoy reading anymore, because she finds her mind drifting toward her worries instead of the words on the page.”*

This was then followed by the stigma and help-giving intention items described below:

Stereotypes: The measure of the WNS stereotype consisted of three items adapted from Griffiths et al.’s Personal Depression Stigma Scale [50]. Participants rated statements such as “People with a problem like Katie’s could snap out of it, if they wanted” on a five-point scale from “strongly disagree” to “strongly agree”, such that higher scores indicated greater endorsement of the stereotype. Responses to the three WNS items were summed and averaged to produce a mean WNS stereotype score. Internal consistency for the WNS items was acceptable, with a Cronbach’s alpha of 0.7.

Prejudice: The prejudice measure consisted of a nine-item emotional-ratings scale used and validated by Angermeyer and Matschinger [51] which included three subscales measuring anger, fear, and pity. Each subscale consisted of 3 items. Participants rated their agreement on a five-point scale from strongly disagree to strongly agree to statements such as “Katie’s behaviour makes me feel afraid”. Higher scores on each subscale indicate more anger, more fear, and more pity. Internal consistency values were 0.7 for the anger subscale, 0.75 for the fear subscale, and 0.4 for the pity subscale.

Discrimination: The discrimination measure used was Kelly and Jorm’s [52] social distance scale. This consists of six items relating to participants’ willingness to engage in contact with a hypothetical peer (e.g., going to the peer’s house after school), on a four-point scale (‘definitely unwilling’ to ‘definitely willing’). These items were then reverse scored and summed so that higher scores indicate a higher desire for social distance. Reliability for this scale has previously been reported as 0.9 [52]. Internal consistency for the present study was 0.85.

Help-giving intentions: Participants were also asked to rate the likelihood that they would offer to help the peer with their problem, on a five-point scale from ‘very unlikely’ to ‘very likely’. This item was adapted from measures of help-giving intentions used in previous research, such as Cavallo, Zee and Higgins [53].

### 2.3. Procedure

Ethical approval was obtained from the Trinity College Dublin School of Psychology Research Ethics Committee (approval code SPREC042018-1). Permission to conduct the study was then obtained from each school and the youth group. Informed consent from a parent or guardian was obtained via an information and consent form sent home with students prior to the commencement of the study. On the day of data collection, those students with signed parental consent forms who wished to participate were given their own information and consent form to sign. Participants were then presented with a pen-and-paper survey.

### 2.4. Data Processing

Descriptive statistics, including percentages, means (*M*), and standard deviations (*SD*) were used to assess if adolescents endorse the WNS stereotype for hypothetical peers presented with symptoms of GAD. Pearson’s *r* correlations were conducted to determine the relationship between the WNS items and, when these items were combined to form a WNS subscale, mean score differences for males and females were compared using independent *t*-tests. These calculations were done using SPSS version 25 (IBM, Armonk, NY, USA). The PROCESS (Version 3) macro [54] for SPSS generated a multiple mediator model to explain how adolescents’ endorsement of the WNS stereotype was related to aspects of prejudice, discrimination and help-giving responses.

## 3. Analysis and Results

### 3.1. Do Adolescents Endorse the ‘Weak-Not-Sick’ (WNS) Stereotype for Hypothetical Peers Presented with Symptoms of GAD?

The majority of participants, almost two thirds, indicated ‘disagree’ or ‘strongly disagree’ with reference to the three statements that tapped into the WNS stereotype. Approximately one fifth of the sample neither agreed nor disagreed with the statements, leaving between 11.5% and 17% of adolescents who endorsed the stereotype. Exact values are detailed in Table 1.

Pearson’s *r* correlations showed that scores for each item moderately correlated with each other, with a range of 0.40 to 0.42 (*p* < 0.01). When scores for the three items were added and averaged to create the WNS stereotype subscale (Cronbach’s alpha = 0.67), the mean value was 2.15 (SD = 0.88), within a range from one to five, where lower scores reflect less endorsement of the stereotype. Independent *t*-tests to investigate differences in the mean score for males and females on this subscale indicated that adolescent boys (*M* = 2.33, *SD* = 0.91) were significantly more likely than adolescent girls (*M* = 2.07, *SD* = 0.86) to endorse the view that the vignette character with GAD was WNS (*t*(229) = 2.08, *p* < 0.05, effect size Cohen’s *d* = 0.3).

### 3.2. Does Greater Endorsement of the ‘Weak-Not-Sick’ Stereotype for Adolescent Peers with GAD Relate to Greater Prejudice and Discrimination and Less Help-Giving Intentions?

**Figure 1:**
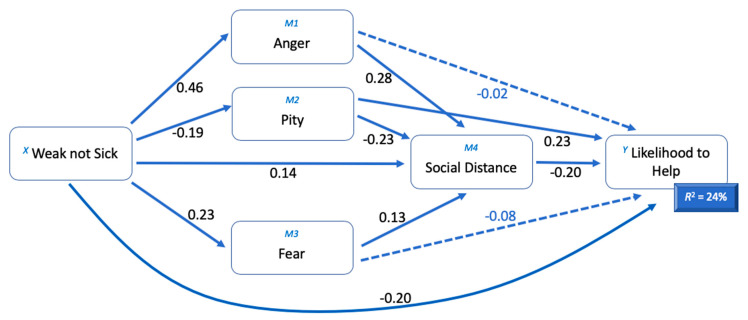
Anger, pity, fear and social distance as mediators in the relationship between the ‘weak-not-sick’ stereotype and help-giving intentions. Standardized path coefficients are presented. Total effect: *b* = −0.3425, *SE* = 0.0641, *p* < 0.0000. Direct effect: *b* = −0.2036, *SE* = 0.0694, *p* < 0.05. Indirect effect: XM_1_Y = −0.0082, Boot*SE* = 0.0380, 95% Confidence Interval (CI) = −0.0893 to 0.0625; XM_2_Y = 0.0428, Boot*SE* = 0.0229, 95% CI = −0.0963 to 0.0075; XM_3_Y = −0.0194, Boot*SE* = 0.0148, 95% CI = −0.0527 to 0.0062; XM_4_Y = −0.0279, Boot*SE* = 0.0178, 95% CI = −0.0685 to 0.0005; XM_1_M_4_Y = −0.0257, Boot*SE* = 0.0126, 95% CI = −0.0550 to −0.0058; XM_2_M_4_Y = 0.0087, Boot*SE* = 0.0057, 95% CI = 0.0225 to 0.0010; XM_3_M_4_Y = −0.0062, Boot*SE* = 0.0041, 95% CI = −0.0162 to −0.0003, *N* = 221.

A multiple mediator model was developed to explore how endorsing the WNS stereotype for a hypothetical peer with symptoms indicative of Generalised Anxiety Disorder had a direct negative relationship with the likelihood to offer help and an indirect association through the mediating prejudice variables of anger, pity and fear and a measure of desired social distance. Table 2 displays descriptive information and the bivariate relationships among key variables and Figure 1 displays the proposed model.

Overall, the model accounted for 23.53% of the variance in adolescents’ likelihood to help (*R*^2^ = 0.2353, *F* (5, 215) = 13.23, *p* < 0.0000). The direct effect of the WNS stereotype on helping intentions was significant (*b* = −0.2036, *SE* = 0.0694, *p* < 0.05), so that greater endorsement of the stereotype was associated with less likelihood to help. The WNS stereotype was also significantly associated with less pity (*b* = −0.1882) and greater anger (*b* = 0.4584), fear (*b* = 0.2343) and a desire for social distance (*b* = 0.1407). Pity was significantly associated with less social distance (*b* = −0.2322) and greater intention to help (*b* = 0.2273), while anger (*b* = 0.2830) and fear (*b* = 0.1326) were both significantly associated with greater social distance, but were not significantly associated with intention to help. Social distance was significantly associated with less intention to help. As such, significant indirect effects were also noted in the relationship between WNS and intention to help. Pity (*b* = 0.0428, *SE* = 0.0229, 95%CI: 0.0963 to 0.0075) and social distance (*b* = −0.0279, SE = 0.0178, 95%CI: −0.0685 to −0.0005) independently partially mediated the relationship between the WNS stereotype and likelihood to help. Further partial mediation was provided through anger combined with social distance (*b* = −0.0255, SE = 0.0124, 95% CI: −0.0550 to −0.0059), pity with social distance (*b* = 0.0086, SE = 0.0055, 95% CI: 0.0216 to 0.0010), and fear with social distance (*b* = −0.0061, SE = 0.0041, 95% CI: −0.0160 to −0.0003). Other indirect effects were not significant. Table 3 contains the model coefficients.

## 4. Discussion

The results of the present study found that endorsement of the WNS stereotype was present among a substantial proportion of the sample. While it is positive that approximately two thirds of participants disagreed with the WNS stereotype, 11–17% explicitly endorsed the WNS stereotype across each of the three items that comprise it. Additionally, the one fifth of participants who chose the neutral “neither agree nor disagree” option across the three items cannot be said to reject the WNS stereotype.

This is a broadly similar proportion to that found in some previous studies involving social phobia, in which 15–22% of participants aged 15–25 endorsed the various WNS items [55], although lower than that found in others that examined social phobia stigma [39]. Further studies are needed in the context of GAD to establish whether the proportion of students found to endorse the WNS stereotype in the present study is generalisable to the adolescent population at large. If it is, this represents a major target for stigma reduction efforts.

The study also found that male adolescents were significantly more likely to endorse the WNS stereotype than females. This is in line with previous research from the broader stigma literature [48,49] and suggests that adolescent males may be one group of interest when developing future educational interventions.

The present study also demonstrated that greater endorsement of the WNS stereotype was associated with significantly higher prejudice (higher fear, higher anger, and less pity), significantly higher levels of desired social distance, and significantly lower likelihood of helping the person with GAD. These findings are significant, as they shed light on the potential pathways between stigma and help-giving, specifically with regard to the WNS stereotype, which has not been studied in detail in anxiety disorders, or in the context of adolescence to date. The results show both a direct relationship between WNS and help-giving, and an indirect relationship between WNS and help-giving mediated by prejudice (anger, fear and pity) and desired social distance.

The direct association between WNS and help-giving may relate to perceptions of the need for help; if a person believes that symptoms of GAD are indicative of a personal weakness rather than a serious mental illness, they may then not perceive the problem as being one that necessitates help or intervention in general. This has been implied in research into help-seeking for anxiety disorders in which minimisation and misperception of symptoms as being normal, everyday stress is associated with low rates of help-seeking by people with anxiety disorders [11] but has yet to be investigated with regard to help-giving responses. Future research should attempt to parse this relationship further.

The indirect pathway between the WNS stereotype and help-giving shown in Figure 1 (above) endorses the tripartite model of stigma proposed by Corrigan and Watson [22], in that the relationship between stereotypes (WNS), prejudice and discrimination are in the expected direction, with greater endorsement of negative stereotypes leading to higher levels of prejudice and greater discrimination. Specifically, endorsement of the WNS stereotype was associated with higher prejudice (greater levels of anger and fear, and less pity), which in turn was associated with higher levels of discrimination in the form of desire for social distance. This is in line with previous research and discussion of the relationship between stigma components in general [22,56].

In essence, the results show that if participants perceive symptoms of GAD as being the vignette character’s own fault, they are less likely to feel sorry for them, are more likely to feel negative emotions such as anger and fear, and are less likely to want to spend time with them. These factors (higher prejudice and discrimination) in turn are associated with less reported likelihood that participants would help the person experiencing GAD. These findings are among the first to examine the relationship between endorsement of the WNS stereotype and help-giving, in the context of adolescent GAD, and the first, to our knowledge, to examine the mediating factors underlying that relationship.

While research into these underlying processes are severely lacking in the mental illness stigma literature, these results are supported by findings from the broader psychological literature on the role of attributions and help-giving responses, which have found that when people are perceived to be in control of their own negative actions or experiences, this is associated with increased anger, less pity, and a lower likelihood of helping responses [57]. A greater focus on these underlying pathways in the context of stigma in general, and anxiety stigma in particular, is needed in future research, both to validate existing models of stigma, and to shed light on new targets for stigma reduction efforts.

The study also found that the WNS stereotype was directly associated with increased desire for social distance, which replicates findings from previous studies, as outlined in a review by Kaushik et al. [44]. Why participants’ perceptions of personal weakness are associated with higher desired social distance independently of (as well as mediated by) the prejudice items measured is unclear, although it is possible that perceptions of personal weakness may lead to some other affective reaction that is not captured by the standard prejudice measure used in this study, which may mediate the relationship in a similar way to that of anger, pity and fear. Additionally, pity was found to be associated with increased likelihood of offering help independently of social distance, unlike anger and fear. This finding is supported by previous research which has shown that sympathy is a consistent predictor of help-giving intentions [57,58].

### Implications, Limitations and Directions for Future Research

The results of this study indicate that a proportion of adolescents endorse the WNS stereotype toward GAD, and that endorsement of this stereotype is associated with prejudice, discrimination, and help-giving intentions. These findings have implications both in terms of providing a clear target for stigma reduction efforts, and for potential interventions aimed at increasing help-giving intentions among adolescents toward their peers with GAD and other anxiety disorders. By educating adolescents as to the serious nature of GAD, in terms of its severity and impact on those affected [12,13], as well as aiming to increase empathy and sympathy for those with the condition, it is possible that both stigmatising and help-giving responses could be targeted simultaneously. Given the emergence of anxiety disorders such as GAD in adolescence, and their potential to become chronic, lifelong conditions [5,13,16], early intervention opportunities such as these must be investigated and capitalised on. Additionally, given the relationship between stigma and treatment-seeking seen in previous research, stigma-reduction efforts also have implications for uptake of appropriate treatment for anxiety disorders, which is currently extremely low [9,24,25,26].

Limitations of the study include a relatively small sample size that was limited in scope to older adolescents. In addition, the low Cronbach’s alpha value for the Pity subscale in the present study suggests that related findings should be interpreted with caution. Finally, there is a potential for social desirability bias when using explicit measures of stigma [59], and our measures therefore may not be capturing a portion of stigmatising responses. As such, future research should expand their focus to a wider age range of adolescents, with larger sample sizes, in order to increase the generalisability of the findings and investigate whether they hold true in different age groups. Future studies should also consider adding implicit measures of stigma in order to reduce any potential social desirability bias. Additionally, future research should examine the relationship between the WNS stereotype and help-giving, and underlying mediating factors, in other anxiety disorders, which have also been neglected in previous research.

## 5. Conclusions

Adolescence represents an important opportunity for early intervention in clinical anxiety disorders, including GAD, which is particularly likely to have a long delay between onset of symptoms and initiating treatment. Mental illness stigma has been implicated in negative outcomes across a range of mental illnesses, including GAD, but anxiety disorders in general have been neglected in the stigma literature. Early research suggests that anxiety stigma in particular may focus on a perception that symptoms are due to personal weakness. The present study examined the WNS stereotype in the context of GAD and found it to be endorsed by a significant minority of adolescents. The study also outlines a model for how anxiety stigma may relate to help-giving, and demonstrates the significant associations between endorsement of the WNS stereotype and prejudice, discrimination, and help-giving intentions. In addition to adding to the limited knowledge base on the nature of anxiety stigma in adolescents, the study then provides, via the WNS stereotype, a specific target for general stigma-reduction interventions with additional implications for help-giving responses toward those with GAD.

## Figures and Tables

**Table 1:** Adolescents’ endorsement of items comprising the ‘weak-not-sick’ stereotype.

Stereotype Statement	*M* (*SD*)	Achieved Range	Disagree or Strongly Disagree	Neither Agree nor Disagree	Agree or Strongly Agree
“…could snap out of it if they wanted”	2.26 (1.16)	1–5	64.8%	18.2%	17%
“…a sign of personal weakness”	2.12 (1.12)	1–5	65.8%	19.6%	14.6%
“…not a real medical illness”	2.12 (1.11)	1–5	65.5%	23%	11.5%

**Table 2:** Descriptive details for, and correlations between, key variables.

Variable	*M* (*SD*)	Achieved Range	Weak Not Sick	Anger	Pity	Fear	Social Distance	Likelihood to Help
Weak not Sick	2.15 (0.88)	1–5	1	0.441 **	−0.181 *	0.215 **	0.347 **	−0.332 **
Anger	4.65 (1.95)	3–13		1	−0.268 *	0.366 **	0.429 **	−0.284 **
Pity	11.90 (1.91)	3–15			1	−0.054	−0.337 **	0.330 **
Fear	5.82 (2.32)	3–12				1	0.250 **	−0.203 *
Social distance	11.03 (3.86)	6–24					1	−0.345 **
Likelihood to help	4.39 (0.891)	1–5						1

* *p* is significant at the 0.05 level (one-tailed) ** *p* is significant at the 0.01 level (one-tailed).

**Table 3:** Model coefficients for the effect of weak not sick on likelihood to help with anger, pity, fear and social distance as mediators.

Variable	Anger			Pity			Fear			Social Distance			Likelihood to Help		
	Coeff.	*SE*	*p*	Coeff.	*SE*	*p*	Coeff.	*SE*	*p*	Coeff.	*SE*	*p*	Coeff.	*SE*	*p*
Weak not sick	0.4584	0.0583	0.0000	−0.1882	0.0655	0.0045	0.2345	0.0642	0.0003	0.1407	0.0643	0.0298	−0.2036	0.0694	0.0037
Anger										0.2830	0.0693	0.0001	−0.0180	0.0767	0.8150
Pity										−0.2322	0.0590	0.0001	0.2273	0.0652	0.0006
Fear										0.1326	0.0621	0.0338	−0.0827	0.0669	0.2177
Social distance													−0.1983	0.0726	0.0068
	*R^2^* = 0.2200 *F* (1219) = 61.78 *p* = 0.0000			*R^2^* = 0.0363 *F* (1219) = 8.258 *p* = 0.0045			*R^2^* = 0.0573 *F* (1219) = 13.32 *p* = 0.0003			*R^2^* = 0.2911 *F* (4219) = 22.18 *p* = 0.0000			*R^2^* = 0.2353 *F* (5219) = 13.23 *p* = 0.0000		
